# Protection Management, Nurse Plants, and Diversity of Companion Species Influence Natural Regeneration of *Pinus gerardiana* in the Western Himalayan Pine Forests

**DOI:** 10.1002/ece3.73187

**Published:** 2026-07-01

**Authors:** Atiqullah Sultani Ahmadzai, Hamid Ejtehadi, Maral Bashirzadeh, Mohammad Farzam

**Affiliations:** ^1^ Quantitative Plant Ecology and Biodiversity Research Lab., Department of Biology, Faculty of Science Ferdowsi University of Mashhad Mashhad Iran; ^2^ Department of Biology Paktia University Paktia Afghanistan; ^3^ Department of Plant Biology, Faculty of Basic Sciences University of Mazandaran Babol Iran; ^4^ Department of Range and Watershed Management, Faculty of Natural Resources and Environment Ferdowsi University of Mashhad Mashhad Iran; ^5^ ARC Training Center for Healing Country, Department of Molecular and Life Sciences Curtin University Perth Australia

**Keywords:** Afghanistan, diversity of companion species, *Pinus gerardiana*, plant–plant interactions, protection management strategy, soil parameters

## Abstract

Protection strategies, nurse plants, and abiotic factors affect the natural regeneration of tree seedlings. However, their interactions have been less considered in restoration and management programs. We assessed the impacts of nurse plants, diversity of companion species, and soil factors on the density of *Pinus gerardiana* seedlings under different protection levels in the alpine pine forests of the western Himalayas in Afghanistan. We selected three sites with different protection strategy levels (low protection (LP), medium protection (MP), and high protection (HP)). Three macro‐plots were established in each site; then, 10 × 10 m^2^ quadrats were established within the macro‐plots to quantify woody species. Then, three 1 × 1 m^2^ quadrats were established within 10 × 10 m^2^ quadrats to measure herbaceous species. Soil factors were collected within the quadrats. In each site, some dominant woody species were selected as nurse species, and the area beneath their canopies was sampled using 0.5 × 0.5 m^2^ quadrats and the same number of quadrats in open areas to assess the density of pine species without the effects of woody species. Linear mixed‐effect models and variation partitioning analysis were performed to assess the effects of the predictors on pine density. The density of pine exhibited a direct relationship with the presence of nurse species, particularly in HP and MP sites. A negative correlation was found between the diversity of companion species and pine density in LP and MP sites, whereas a positive effect of such diversity was found on pine density in the HP site. Phosphorus and sulfur were identified as primary soil factors influencing pine density in HP and MP sites, while organic carbon and pH were important to explain pine density in the LP site. Overall, the diversity of companion species, nurse plants, and soil parameters significantly affected the density of pine. However, their effects were overshadowed by management strategies.

## Graphical abstract

Studies examining the factors influencing the population density of keystone plant species offer valuable insights for forest management and conservation efforts. Utilization level, nurse plants, and abiotic factors significantly affect the natural regeneration of juvenile trees. However, their interactions have been less frequently considered in restoration and management programs. In this research, we evaluated the impacts of some protection management strategies, nurse plants, plant community diversity, soil factors, and their interactions on the density of *Pinus gerardiana* juveniles in the alpine pine forests of the western Himalayas in Afghanistan.

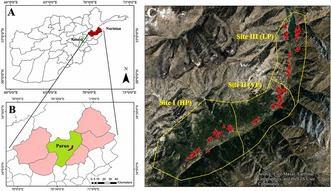

## 1 Introduction

Understanding the factors that influence natural regeneration is essential for conserving keystone tree species such as *Pinus gerardiana* . Protection management, plant–plant interactions, and diversity of companion species are key determinants of regeneration dynamics, as they mediate biotic interactions, microclimatic conditions, and resource availability. Vegetation characteristics including floristic diversity and structural attributes of dominant species (e.g., density and abundance) provide critical insights into how biotic and abiotic factors, along with human management practices, affect seedling establishment and survival (Singh 2002; Gosain et al. 2015; Baboo et al. 2017; Fartyal et al. 2022). Studying these vegetation parameters thus provides essential information for understanding regeneration patterns in *P. gerardiana* forests and for guiding effective conservation and restoration strategies (Phillips et al. 2003; Bisht et al. 2022; Gordon and Newton 2006a, 2006b).

The persistence of a species within forest communities largely depends on successful regeneration under variable environmental conditions (Bargali and Bargali 2016). Seedling and sapling population dynamics serve as important indicators of the regeneration potential and demographic status of individual species (Mourya et al. 2019). Considerable evidence on natural regeneration in pine forests has been gathered through active restoration programs and studies evaluating the effectiveness of human management strategies (Crouzeilles et al. 2016; Djenontin et al. 2018; Souza‐Alonso et al. 2022; Bashirzadeh et al. 2023, 2024). However, limited information exists regarding the roles of vegetation characteristics—such as plant community diversity, tree density of mature individuals, and biotic interactions, including passive restoration approaches—in influencing seedling regeneration.

The relationship between the diversity of companion species—defined as plant species that co‐occur with a focal tree species—and the density of that tree species has been widely studied in plant ecology. A large body of evidence indicates a predominantly negative relationship, whereby increasing companion species diversity promotes competition for limiting resources, leading to reduced density of the tree species at large spatial scales (i.e., the areas greater than 1 m^2^) (Adler et al. 2018). However, the strength and direction of this relationship are highly context‐dependent. Environmental conditions, such as resource availability and abiotic stress, the type and intensity of management strategies, as well as presence of nurse plants, those provides a suitable microenvironment beneath canopies to support less tolerant species, can modify the relationships between diversity of companion species and density of tree species, resulting in variable effects of companion species diversity on tree species density (Levine et al. 2017).

Plant–plant interactions play a pivotal role in the succession and restoration of natural and degraded ecosystems, particularly under moderate to extreme environmental conditions (Soliveres and Maestre 2014; Soliveres et al. 2014, 2015; Bashirzadeh, Shefferson, and Farzam 2022; Bashirzadeh, Soliveres, et al. 2022; Rafiee et al. 2023). Nurse plants can improve target plant establishment and minimize negative impacts in restoration projects through “precision forest restoration” particularly at small spatial scales (i.e., less than 1 m^2^) (Castro et al. 2021). They enhance the survival, growth, and fitness of less tolerant species by creating favorable microenvironments beneath their canopies, mitigating harsh conditions across multiple spatial scales (Weltzin and McPherson 1999; Callaway 1995; Castro et al. 2002; Padilla and Pugnaire 2006). The facilitative effects of nurse plants are context‐dependent, varying with species life form, habitat, and human interferences, and are strongest under intermediate to severe environmental conditions (Bashirzadeh, Shefferson, and Farzam 2022; Bashirzadeh, Soliveres, et al. 2022; Bashirzadeh et al. 2023). In natural and degraded ecosystems, nurse shrubs often provide the most pronounced facilitation due to morphological traits such as low root‐to‐shoot ratios, which reduce competition and enhance plant diversity, whereas nurse trees and grasses tend to exert narrower facilitative effects under moderate conditions (Gómez‐Aparicio 2009; Lu et al. 2017).

Soil parameters constitute another major abiotic driver of natural regeneration, influencing ecological processes, nutrient availability, and forest productivity (Manral et al. 2020; Fartyal et al. 2025). Strong relationships exist between soil physicochemical characteristics and vascular plant diversity (Marini et al. 2007; Kolahi and Atri 2014), highlighting their role in forest regeneration (Chazdon et al. 2016; Bryan‐Brown et al. 2020). Key soil nutrients such as nitrogen, phosphorus, and potassium, along with pH and organic matter, directly affect tree growth, seedling survival, and community composition (Binkley 1986; Deshmukh 2012; Kekane et al. 2015; Colmanetti et al. 2021; Poggio et al. 2021). Despite this knowledge, the relative importance of soil parameters compared with biotic drivers, such as community diversity and nurse plants, remains poorly understood in pine forests.

*Pinus gerardiana* is a culturally and economically important tree species in Afghanistan, India, and Pakistan, occurring at altitudes between 1800 and 3350 m in the western and eastern Himalayas. It provides vital resources, including food, fuelwood, medicinal plants, pasture, and animal shelter, and contributes significantly to ecosystem structure and function (Singh et al. 1973; Farjon 1998; Ahmed and Latif 2007; Malik and Shamet 2008; Khan et al. 2015; Kumar et al. 2016). In Afghanistan, particularly in Nuristan, local tribes manage forest utilization through measures such as monitoring, restricting pinecone collection, controlling livestock grazing, and regulating firewood harvest (Kuhn et al. 2006; Shalizi and Khurram 2016). Broader institutional frameworks, including NEPA, UNEP, and MAIL, support community‐based forest management and environmental education programs (Qazizada et al. 2021).

Building on global and regional studies of forest regeneration, this study examines how biotic factors (plant–plant interactions and diversity of companion species), abiotic factors (soil properties), and human management strategies (protection levels) interact to influence the natural regeneration of *P. gerardiana* . Although prior research has addressed regeneration drivers across ecosystems, the combined influence of vegetation characteristics and protection management on pine seedling establishment remains underexplored. This study aims to test two hypotheses: (1) the regeneration of *P. gerardiana* seedlings is shaped by interactions among soil properties, diversity of companion species, and plant–plant interactions and (2) anthropogenic impacts associated with forest protection and utilization overshadow other biotic or abiotic factors in determining seedling regeneration. The results of this research will provide practical insights for conservation and restoration and contribute to a broader understanding of plant community ecology.

## 2 Materials and Methods

### 2.1 Study Area

**FIGURE 1:**
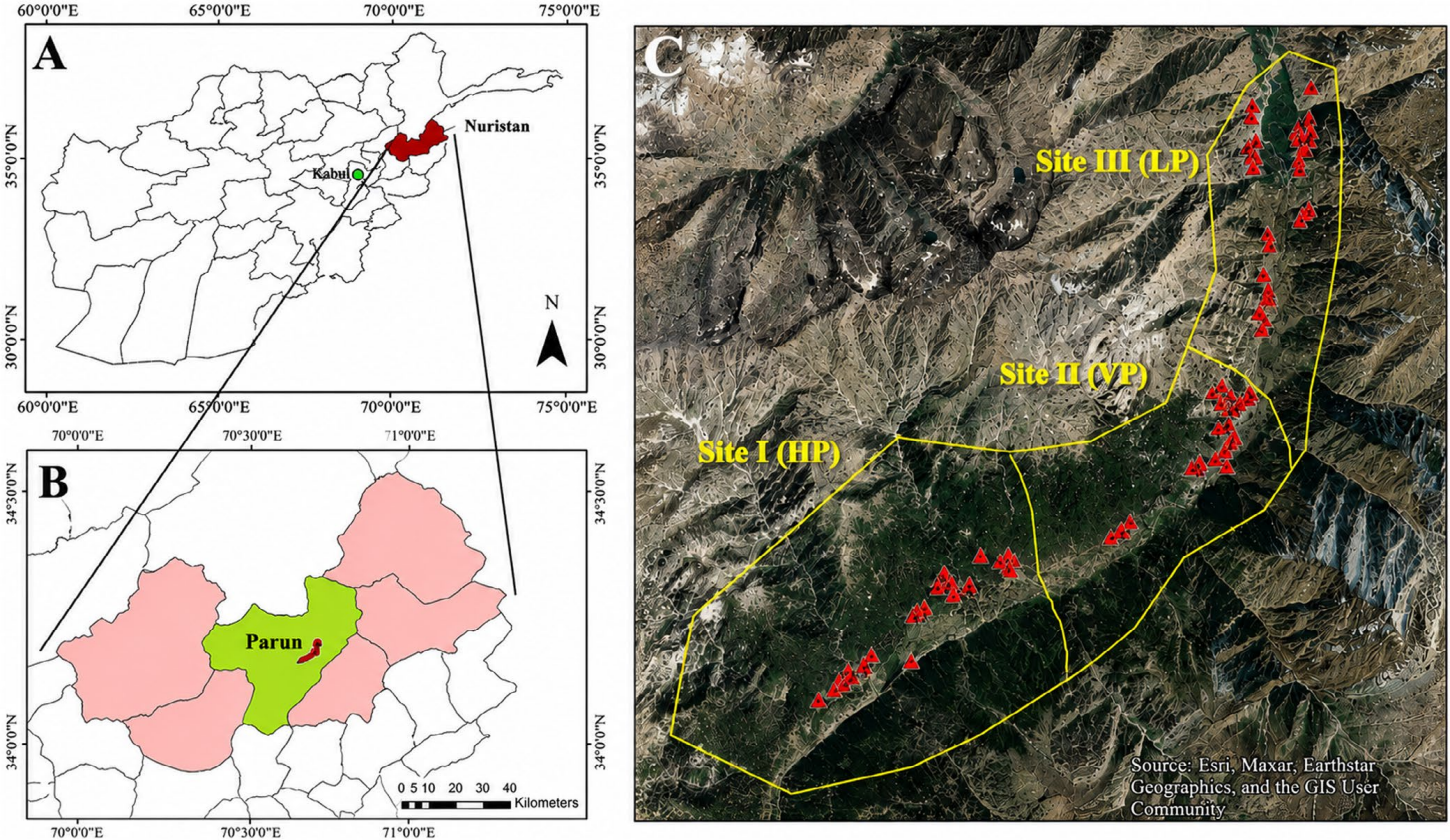
(A) Map showing the provinces' administrative divisions in Afghanistan. (B) The province of Nuristan and the study location represented by the green color. (C) The sites studied based on different levels of protection strategies made in the pine forests of Nuristan province in Afghanistan.

The study was conducted in the pine forests of Nuristan province, Afghanistan, located in the alpine regions of the western Himalayas (between 35°30′82″ N and 35°37′54″ N, and 70°85′82″ N and 70°92′98″ E) (Figure 1). These forests are influenced by Indian monsoons, which bring temperatures ranging from −15°C to 28°C, high wind speeds, and snowfall (Breckle 2007; Breckle and Rafiqpoor 2010, 2022). Situated at an average altitude of 2400 m, the climate is cold and humid, with brief summers (June to August) and long winters (October to April) that include snowfall (Breckle and Rafiqpoor 2022).

### 2.2 Data Collection

#### 2.2.1 Vegetation Sampling

**FIGURE 2:**
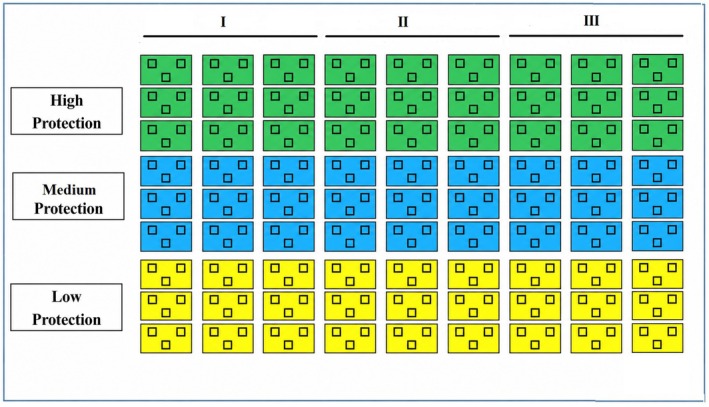
A schematic plan of vegetation sampling based on a systematic‐random procedure in the pine forests of Nuristan, Afghanistan. Three sites were considered based on protection strategies found (i.e., high protection site, medium protection site, and low protection site). Three macro‐plots were established within each site, and nine 10 × 10 m^2^ quadrats were placed within each macro‐plot to assess abundances of woody species. Within each 10 × 10 m^2^ quadrats, three 1 × 1 m^2^ quadrats were established to assess the abundances of herbaceous species.

We considered three categories of protection strategies found in the pine forests of Nuristan province: (i) high protection (HP), (ii) medium protection (MP), and (iii) low protection (LP) in order to design vegetation sampling procedure and assess effects of protection strategies on natural regeneration of the pine forests (Figure 1). HP represents forest sites that are characterized by minimal human modification and are largely free from anthropogenic pressure. In HP sites, strict conservation measures are implemented regarding cone collection, timber harvest, and livestock grazing, where local inhabitants are allowed to cut down only dried‐up trees for fuel use in the summer, and animals are permitted to graze from March to April. This site often provides higher management of ecological benefits, such as carbon sequestration, biodiversity conservation, and the maintenance of watershed health. MP shows forest sites that have experienced some human impacts but are still managed with conservation objectives in mind; these areas often allow certain activities such as controlled logging, which is aimed at balancing ecological integrity with economic benefits. In the MP site, pinecone harvesting and livestock grazing are moderately controlled, but the rules are not clearly defined or strictly enforced. LP exhibits forest sites which are not significantly impacted by human activities and often require careful management to strike a balance between ecological, economic, and social needs. Therefore, in the LP site, there are no restrictions on timber harvesting, cone collection, or livestock grazing. To survey the vegetation within the sites selected, three macro‐plots were established in each site. The distance between macro‐plots was relatively 2–3 km. We set three macro‐plots in each site to ensure the accuracy of the survey data. Then, nine 10 × 10 m^2^ quadrats were established in each macro‐plot to quantify the abundances of woody species using cover‐based measurements. Within each 10 × 10 m^2^ quadrat, three 1 × 1 m^2^ plots were placed at two corners and at the center of the quadrat to quantify the abundances of herbaceous species using cover‐based measurements (Figure 2). The majority of the 10 × 10 m^2^ quadrats and their corresponding 1 × 1 m^2^ quadrats were established on north‐facing slopes with a slope degree of 25 as mean values and at an altitude ranging from 2300 to 2700 m (for more information, see Table S1). Therefore, there is no significant difference among the sites based on environmental conditions. We pooled 1 × 1 m^2^ quadrats with their corresponding 10 × 10 m^2^ quadrats to build a community including both woody and herbaceous species. Overall, 257 plant species, comprising 176 genera and 61 families, were identified in all the sites.

### 2.3 Plant–Plant Interactions

To investigate the effects of plant–plant interactions on pine density, we firstly determined dominant woody species at each site. We found shrub species *Cotoneaster nummularius* Fisch. & C.A. Mey., and the mature target tree *P. gerardiana* Wall. ex D. Don as two dominant woody species in the sites. We considered these woody species as potential nurse and 30 0.5 m × 0.5 m quadrats were surveyed under the canopies of these dominant nurse species, and the same number of quadrats in open areas far from such potential nurse. Open areas were those located > 2 m away from any individuals of the selected nurses, for trees in which case open areas were located at a distance equal to the canopy width (Soliveres and Maestre 2014). Then, density of juvenile pine trees was measured beneath the canopies of nurse species and their open areas. Juvenile pines were defined as young trees shorter than two meters in height. Moreover, we measured density of juvenile pine under presence of both two nurse species (i.e., *C. nummularius* /adult pine), each occupying a different vertical stratum of pine forests (for more information see Table S2).

### 2.4 Soil Variables

To measure soil physicochemical properties at each site, the soil samples (~750 g) were taken at the center of each 1 m × 1 m quadrats established with a depth of 0–5 cm. The soil samples were placed in a polyethylene bag, labeled, and transported to the laboratory to measure pH, electrical conductivity (EC), organic carbon (OC), sodium (Na), total nitrogen (N), potassium (K), phosphorus (P), and soil texture components, including lime, silt, sand, and clay percentage. The Bykas hydrometric method 110 (Bouyoucos 1962) was used to determine soil texture. Total nitrogen (N) was determined by the Kjeldahl method. Organic carbon (OC) was analyzed by the Walkley and Black (1934) method (Nelson and Sommers 1996). Soil electrical conductivity (EC) and acidity (pH) were determined using pH and EC meters. Total sodium (Na) was analyzed by Fame Atomic Absorption Spectrophotometer (Beckett 1964). Absorbable phosphorus was analyzed by the Olsen method. The percentage of total lime was measured by the titration method with 0.01 NaOH (Barrow and Cox 1990). Finally, we prepared a matrix of 12 independent variables (i.e., sodium [Na], potassium [K], pH, electrical conductivity [EC], lime, total nitrogen [N], phosphorus [P], organic carbon [OC], clay, sand, and silt) and used it in further analyses (for more information see Table S3).

### 2.5 Measuring the Diversity of Companion Species

Species diversity of companion plant species, those present in 10 × 10 m^2^ quadrats with pine species, was measured using the first two RaoQ indices to estimate species richness (*q* = 0) and the exponential of Shannon's entropy (*q* = 1, referring to Shannon's diversity). The Hill number of *q* = 1 is defined as follows:

$$q_{D}=\left\{\begin{array}{ll} {\left(\sum_{i=1}^{S}p_{i}^{q}\right)}^{\frac{1}{1-q}}, & q\neq 1\\ \exp\left(-\sum_{i=1}^{S}p_{i}\ln p_{i}\right), & q=1 \end{array}\right.$$

where *q*_*D*_ are Hill numbers of order *q* or the effective number of species, *S* is the number of species in the sample, and *P*_*i*_ is the proportional abundance of the *i*th species. We calculated Hill numbers, *q* = 0 (species richness) and *q* = 1 (referring to the exponential of Shannon diversity) from the species abundance data of companion species to pine for each 10 × 10 m^2^ quadrat (Hsieh et al. 2016). The calculation was based on the coverage of species, which is less affected by differences in total sampling effort than other methods (Chao and Jost 2012). Hill numbers (*q* = 0 and *q* = 1) were calculated using functions of the R package hillR (Chao et al. 2014).

### 2.6 Statistical Analyses

Variation in pine density (response variable) was analyzed in relation to soil parameters, plant–plant interactions, and diversity of companion species and their interactions across three protection management sites. Before performing any further analysis, we analyzed multicollinearity among the soil variables using Pearson correlation analysis and variance inflation factors (VIFs) (function *vif()* in the package “car” [Fox and Weisberg 2007]) and variables with pairwise Pearson correlation coefficient > 0.75 (Dormann et al. 2013; Wheeler and Tiefelsdorf 2005) and VIF scores > 10 were considered to be highly collinear (O’Brien 2007), and were removed from our list of predictors. Finally, all remaining soil predictors, including organic carbon (OC), phosphorus (P), sulfur (S), pH, EC, and silt//clay, were included in our matrix.

The response of pine density in the sites studied was analyzed in relation to the diversity of companion species and plant–plant interactions via linear mixed effect models with taxonomic indices (*q* = 0 and *q* = 1 indices) for companion species and the presence/absence of nurse species (as plant–plant interaction factor) as fixed effect factors and the number of quadrats within the studied sites as a random factor. We compared models with second‐order Akaike information criteria (AIC) and $R_{\mathrm{adj}}^{2}$ values using the function ‘r. squared GLMM’ in the package “MuMIn.”

To understand how and what soil parameters may affect pine density in the sites, we performed multimodel inference analysis (Burnham and Anderson 2004), using the function “dredge” provided in the “MuMIn” R package (Barton 2013). In each site, we considered the possible models with only ΔAIC (difference in AIC between a focal model and the model with the lowest AIC) < 2, and other models remained.

Finally, to examine the relative importance of soil parameters, plant–plant interactions, diversity of companion species and their interactions in explaining variations in pine density in each site, variation partitioning was performed based on partial linear regression using the “varpart” function in the “vegan” R package (Oksanen et al. 2016). The total percentage of variation explained was divided into a unique and shared contribution for four sets of predictors: (i) soil parameters (i.e., Soil), (ii) Plant–plant interactions, (iii) diversity of companion species, and their interactions (i.e., shared environment between these factors).

## 3 Results

### 3.1 The Effects of the Companion Species Diversity on the Diversity of *P. gerardiana*

**FIGURE 3:**
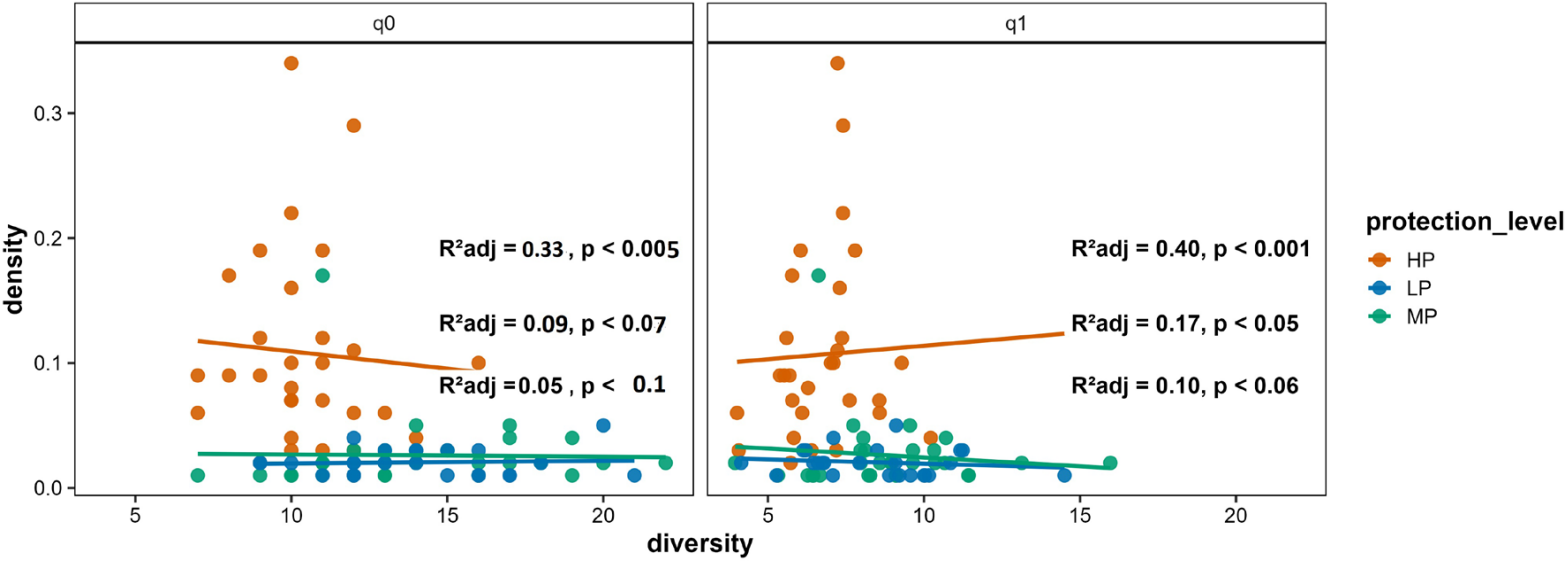
Effects species diversity of companion plant species on the density of *Pinus gerardiana* in different protection management sites (i.e., high protection, medium protection, and low protection).

Protection management and the diversity of companion species significantly (*p* < 0.01) affected density of *P. gerardiana* juveniles (Figure 3). We observed negative effects of species richness of companion plants (i.e., *q* = 0 index) and the diversity of rare companion plants (i.e., q1 index) on the pine density in HP and MP sites, respectively (Figure 3). However, *q* = 0 imposed negative impacts on pine density particularly in HP site (Figure 3; *q*_0_) compared to *q* = 1 index under different levels of protection strategies (Figure 3; *q*_1_). In contrast, positive and significant effects of plant community diversity were found for pine density in MP site (for *q*_0_ index) and HP site (for *q*_1_ index; Figure 3). Interestingly, pine density showed more positive responses to the *q* = 1 index compared to the *q* = 0 index (Figure 3).

### 3.2 The Effects of Plant–Plant Interactions on the Density of *P. gerardiana*

**FIGURE 4:**
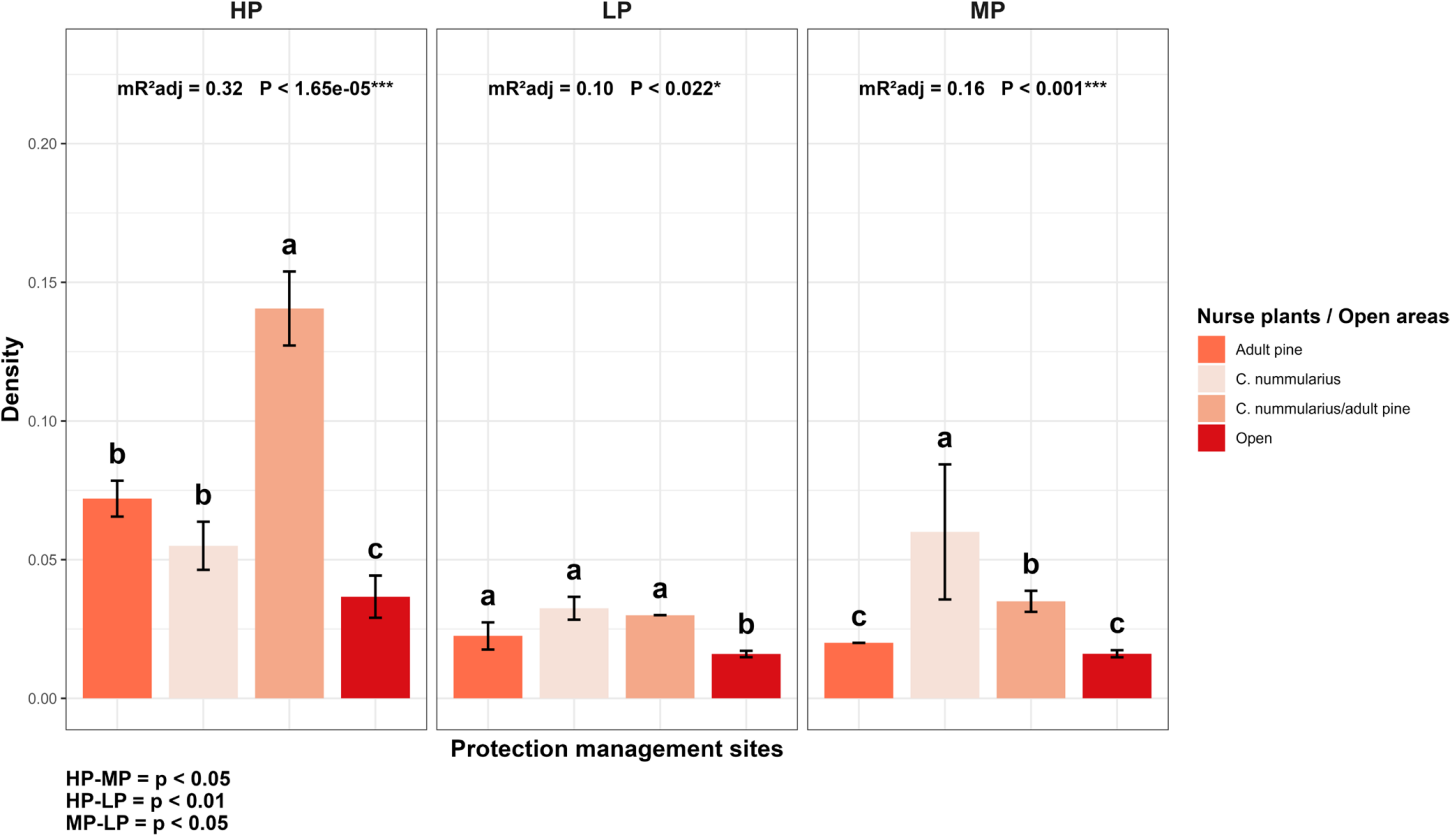
The effects of plant–plant interactions on the density of *Pinus gerardiana* in different protection management sites.

We found plant–plant interactions as a key factor influencing pine density across various protection management sites (Figure 4). Pine density was notably higher beneath nurse shrub ( *C. nummularius* ) in low (LP) and medium (MP) protection sites, and under both *C. nummularius* and adult pine in high protection sites (HP) (Figure 4). In contrast, lower values of pine density were found under open areas in all of the sites studied (Figure 4; see open areas). The outcomes of linear mixed effect models revealed greater effects of plant–plant interactions on pine density in HP sites compared to MP and LP sites (see ${\mathrm{mR}}_{\mathrm{adj}}^{2}$ and *p*‐values in Figure 4). Furthermore, one‐way ANOVA revealed that differences between open areas and nurse species were greater when comparing HP and LP sites than when comparing HP with MP or MP with LP sites (HP–LP: *p* < 0.01; LP–MP and HP–MP: *p* < 0.05). These findings underscore the significant influence of plant–plant interactions on pine density, particularly in HP sites (Figure 4).

### 3.3 Effects of Soil Variables on the Density of *P. gerardiana*

The results of multimodel inference revealed the significant impact of some soil variables on the density of pine depending on the protection levels considered. In this context, phosphorus (P) and sulfur (S) were the primary soil factors influencing the density of pine trees in high protection (HP) sites. Moreover, silt.clay and EC were identified as other key factors affecting pine density in HP sites (Table 1). In the MP site, P, EC, and silt.clay indicated influences on the density of pine tree. Whereas, organic carbon (OC), P, and pH emerged as key soil factors on the density of pine trees in low protection (LP) (Table 1).

**TABLE 1:** Soil parameters contributing to variations in pine density in different sites [i.e., low protection (LP), medium protection (MP), and high protection (HP) sites] evaluated by multimodel inference analysis.

Site	Models	OC	P	pH	S	Silt.clay	EC	df	logLik	AICc	Delta	Weight
HP	Model 1		−0.0194		0.0137			4	42.901	−76.0	0.00	0.446
HP	Model 2					0.0014		3	40.701	−74.4	1.63	0.198
HP	Model 3		**−**0.0178		0.0151		0.0004	5	43.529	−74.2	1.78	0.183
MP	Model 1		0.0075				0.0018	4	49.667	−89.5	0.00	0.192
MP	Model 2		0.0062				0.0013	5	51.132	−85.4	0.11	0.181
MP	Model 3					0.0013	0.0009	4	49.541	−85.3	0.25	0.169
LP	Model 1	−0.0091	−0.0014	−0.108				5	94.534	−176	0	1

### 3.4 Relative Importance of Soil, Plant–Plant Interactions and the Diversity of Companion Species on the Density of *P. gerardiana*

**FIGURE 5:**
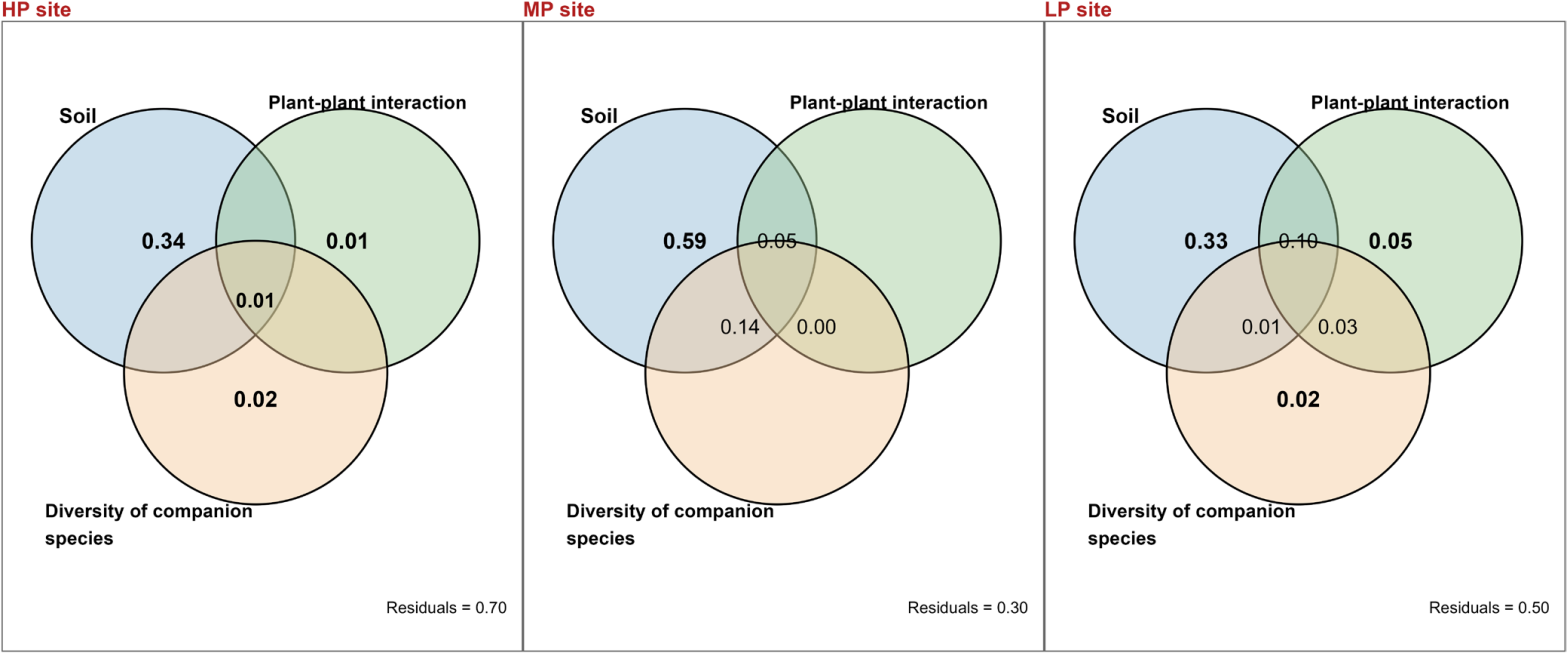
The relative importance of soil factors, plant–plant interactions, and the diversity of companion species on the pine density in different sites studied [i.e., high protection site (HP), medium protection site (MP), and low protection site (LP)] in Nuristan forests.

The amount of variance explained for pine density increased when including soil factors and their interactions with other predictors, particularly plant–plant interactions, in all of the sites studied (Figure 5). This was particularly true for the pure fraction of soil factors which accounted for a substantial share of the variance explained of pine density. Overall, the diversity of companion species was a weak predictor of pine density and only explained a small proportion of the variance in pine density at HP and LP sites (Figure 5). Interaction between plant–plant interactions and soil factors (see shared area between soil and plant–plant interactions fractions) indicated contributions to explaining variation in pine density at LP and MP sites (Figure 5; LP and MP sites). Moreover, we found that interactions between plant–plant interactions and companion species diversity contributed to explaining the variances of pine density at LP sites, as reflected by their shared variance component (Figure 5; LP site).

## 4 Discussion

Our results demonstrate that natural regeneration of *P. gerardiana* seedlings is significantly influenced by all major biotic and abiotic factors examined. Consistent with our first hypothesis, variation in *P. gerardiana* sapling density was associated with the diversity of companion species, plant–plant interactions, and soil nutrient availability. Notably, in support of our second hypothesis, the facilitative effects of nurse plants were markedly stronger in more highly protected forests. However, the relative importance of these factors varied, and their effects were interactive, as discussed below.

### 4.1 Density of Pine Seedlings Correlated With Diversity of Companion Species

Diversity of companion species significantly affected the density of pine depending on the degree of site protection. Accordingly, pine seedling abundance declined when including an increase in species richness of companion plants, suggesting that higher numbers of co‐occurring species may intensify competition for limiting resources. Classical competition theory predicts stronger interspecific competition and potential competitive exclusion in species‐rich communities when niche overlap is high (Tilman 1982, 1994). Empirical studies further indicate that communities dominated by strong competitors often exhibit reduced richness due to competitive exclusion. However, the richness–competition relationship is context‐dependent. In some plant communities, high species richness can reduce direct competition via niche partitioning, resource complementarity, or facilitative interactions (Wang et al. 2019).

Despite reduced richness, overall diversity of companion species (i.e., *q* = 1) showed a positive correlation with pine sapling density, particularly in high protection site. Higher species richness without a corresponding increase in evenness often reflects increased spatial heterogeneity or disturbance, whereas greater evenness is typically associated with more stable conditions (Dornelas 2010; Boch et al. 2013; De Roy et al. 2013; Kuneš et al. 2019; Mancuso et al. 2023; She et al. 2023). These patterns suggest that *P. gerardiana* regeneration is favored under relatively stable conditions with limited anthropogenic pressure.

### 4.2 How Plant–Plant Interactions Enhance the Density of Pine Seedlings

We consistently observed higher densities of *P. gerardiana* juveniles beneath the canopies of the dominant shrub *C. nummularius* and adult pine trees compared with open microsites. This supports extensive evidence that nurse plants create favorable microenvironments by ameliorating temperature extremes, improving soil moisture and nutrient availability, and reducing biotic stress beneath their canopies (LaPlante and Souza 2018; Huang et al. 2023). These facilitative effects result in higher seedling densities and distinct community compositions relative to open areas (Bashirzadeh, Shefferson, and Farzam 2022; Bashirzadeh, Soliveres, et al. 2022). Facilitation is particularly important during early life stages, when seedlings and juveniles are most vulnerable (Byers 2002; Jankju 2013; Fletcher et al. 2017).

Our results further indicate that the identity and importance of nurse plants vary with protection level. In low protection sites, pine seedling density was higher beneath *C. nummularius* than beneath adult pine trees, highlighting the role of pioneer shrubs in supporting regeneration under disturbed conditions (Torres and Renison 2016). Conversely, under higher protection levels, juveniles were more abundant beneath adult pine canopies, suggesting a shift from shrub‐mediated facilitation to conspecific or forest‐structure‐driven regeneration.

These patterns align with the Intermediate Disturbance Hypothesis, which predicts maximum regeneration and diversity under moderate disturbance or management intensity (Huston 2014). Facilitative effects of nurse plants appear strongest under intermediate protection, where stress promotes facilitation but does not prevent recruitment (Beckage and Stout 2000; Dee and Menges 2014). These findings emphasize that nurse shrubs and adult trees play complementary roles in sustaining *P. gerardiana* regeneration across management gradients.

### 4.3 Soil Fertility Provides Context for Nurse Plant Facilitation of Pine Seedlings

In the highly heterogeneous Himalayan landscapes, steep bioclimatic gradients occur over short distances, resulting in pronounced spatial variation in vegetation patterns. Soil physicochemical properties, topography, climate, weathering processes, microbial activity, and interactions with other biotic and abiotic factors strongly shape these patterns (Paudel and Sah 2003; Bargali et al. 2018; Pandey et al. 2024; Fartyal et al. 2025; Garg et al. 2025). In this study, soil parameters—including organic carbon, phosphorus, sulfur, and pH—explained a substantial proportion of variation in pine seedling density, underscoring the importance of soil fertility in regeneration.

Enhanced soil fertility likely promotes pine regeneration by increasing moisture and nutrient availability during early establishment stages (Bhadouria et al. 2016). These conditions may strengthen facilitative interactions by nurse plants, which improve soil organic matter, moisture retention, and nutrient availability beneath their canopies. Fertility‐mediated effects can also indirectly enhance plant community diversity, reinforcing positive feedbacks between soil, nurse plants, and regeneration dynamics (Savva et al. 2010; Fonseca and Duarte 2017; Rafiee et al. 2023).

The significant effects of soil parameters and their interactions with plant–plant interactions on pine density highlight the role of plant canopies in modifying soil fertility (Isichei and Muoghalu 1992; Rand 2004; Acuna‐Rodriguez et al. 2006; Kooch and Dolat Zarei 2023). The effects of soil pH associated with low protection strategies represent altered nutrient availability, impaired uptake, or reduced soil water in this site (Kimberley et al. 2015; Badalamenti et al. 2018).

### 4.4 Interactive Effects of Soil Conditions, Plant–Plant Interactions, and Diversity of Companion Species

Our results underscore the critical importance of management practices, nurse plants, and soil parameters in natural regeneration of *P. gerardiana* . Key findings include the necessity of maintaining soil fertility, preserving nurse plants (including older pines), and enforcing robust forest protection. Management ultimately governs regeneration by creating conditions that allow other factors to operate effectively. Significant effects of companion species diversity and nurse plants on the density of pine were greater in low protection sites than medium sites. It seems that high utilization rates, frequent disturbances, and low stability in low protection sites increased the influence of biotic factors, emphasizing the pivotal role of effective management in promoting regeneration.

## Author Contributions

**Atiqullah Sultani Ahmadzai:** conceptualization (equal), investigation (equal), methodology (equal), project administration (equal), software (equal), writing – original draft (equal). **Hamid Ejtehadi:** conceptualization (equal), funding acquisition (equal), methodology (equal), supervision (equal), writing – review and editing (equal). **Maral Bashirzadeh:** conceptualization (equal), investigation (equal), methodology (equal), software (equal), visualization (equal), writing – original draft (equal), writing – review and editing (equal). **Mohammad Farzam:** conceptualization (equal), funding acquisition (equal), resources (equal), supervision (equal), writing – review and editing (equal).

## Funding

This work was supported by the Faculty of Sciences, Ferdowsi University of Mashhad (3/55972).

## Conflicts of Interest

The authors declare no conflicts of interest.

## Supporting information


**Table S1:** Vegetation database collected from the western Himalayan pine forests located in Afghanistan.

**Table S2:** Supporting information on safe site types and their diversity in the western Himalayan pine forests in Afghanistan.

**Table S3:** Soil database from the western Himalayan pine forests in Afghanistan.

## Data Availability

Data and R scripts are available in Supporting Information files.
